# Selecting species for vineyard inter-row vegetation cover requires consideration of microenvironmental conditions

**DOI:** 10.1371/journal.pone.0319848

**Published:** 2025-03-21

**Authors:** Cristina Pornaro, Stefano Macolino

**Affiliations:** Department of Agronomy, Food, Natural Resources, Animals and Environment, University of Padua, Padua, Italy; University of Education, PAKISTAN

## Abstract

Vegetation between the vineyard rows is considered a service crop due to the many ecosystem services it provides. These benefits mostly depend on the species or mixtures selected. Vineyard can directly impact the microclimate by modifying the amount of energy available for the herbaceous layer affecting parameters such as soil temperature and moisture. Our hypothesis was that changes in inter-row vegetation composition change are driven by micronvironmental condition. A field trial was conducted in a vineyard of Cabernet Sauvignon *Vitis vinifera* L. cultivars, managed organically, in north-eastern Italy, where three blends of grass species (*Shedonorus arundinaceus*, *Lolium perenne,* and *Festuca rubra*) and two grass-legume mixtures were grown in the inter-rows. These were compared with spontaneous vegetation and bare soil regularly tilled. Each type of inter-row cover, including resident vegetation, was subjected to mulching and non-mulching treatments. The study aimed at evaluate the response to microenvironmental conditions of seeded species, mixtures, and spontaneous vegetation in the inter-row spaces of the vineyard. The relative abundance of each species was recorded during the spring after seeding (2019) and during the spring of 2020. Soil compaction, soil temperature, and soil moisture were measured during spring 2020. A significant change in botanical composition occurred two years after seeding according to the type of vegetation. However, some species remained in or spread to specific sections of the inter-row. *Lolium perenne* was more abundant in the southwest and northeast sections of the inter-row, where there was greater soil compaction. Similarly, the abundance of weed species such as *Cynodon dactylon, Setaria italica,* and *Plantago lanceolata*, was related to microenvironmental conditions. Seeded (*Festuca rubra*) or weed species (*C. dactylon*, *Erigeron annuus*, and *Lactuca serriola*) appear to benefit from soil moisture and higher temperatures. Therefore, microenvironment adaptability is the primary factor to consider when selecting species for vineyard inter-row cover.

## Introduction

The vegetation growing in the vineyard alleys is regarded as a service crop due to its ability to deliver a wide array of essential ecosystem services, including soil and biodiversity conservation as well as reduction of cultural practices such as soil tillage, irrigation, and herbicide use [1–4]. The management of vineyard grass cover, therefore, plays an important role in European soil conservation policies, including the Standards of Good Agricultural and Environmental Condition (GAEC) established by Council Regulation No.73/2009. Compared with tilled soil, inter-row vegetation cover has been shown to provide a physical barrier that can significantly reduce sediment runoff, with a reduction positively correlated to the percentage of the soil covered by vegetation [3,4]. In some instances, the inter-row sward provides a supportive environment for the biodiversity of arthropods, which are especially abundant in vineyards with low-intensity soil management and are positively correlated with plant species richness [4]. Planting cover crops is increasingly used to suppress weeds by shading or through competition for nutrients and water [5–7], thus reducing the need for herbicides.

Nutritional competition is the primary challenge to the widespread use of grass cover, particularly where plant available soil water is low during the vegetative development phase of the vines, which can greatly reduce vine vigor and plant production [8], even if recent studies indicate no differences in yields between grassed and tilled soil inter-rows [3,4]. In temperate and humid environments in Europe, permanent grass cover is preferred, while in semi-arid regions temporary cover crops are preferred due to concerns for competition with the vines for soil water. Grapevine plants, as a shrub species, can directly impact the microclimate by modifying the amount of energy absorbed by and released from the inter-row vegetation. It can also affect the regulation of air temperature, humidity, and wind speed, and can create thermal and microclimatic refuges [4]. Several studies have been conducted on the effect of shrub species on their subcanopy microclimate, especially in arid and semiarid regions [9–11]. It has been widely recognized the effects of canopy on decreasing summertime soil temperature [9,10,12,13] and evaporation from the soil surface [11], reducing the solar radiation reaching the ground and weaken air circulation near the soil surface [14]. However, most of these studies have been conducted under natural conditions without the anthropic disturbance typical on vineyard management. Preliminary identification of native vegetation is recommended in order to select the most effective species to seed and the cultivation practices to adopt [15]. Sowing a known species after tillage is the best strategy for managing inter-row vegetation cover [15]. For instance, a barley cover crop was found to be the most efficient management system to control bermudagrass [*Cynodon dactylon* (L.) Pers.] and other weeds in a wine grape vineyard in Raimat (Spain) [15]. The management of inter-row vegetation involves mechanical and biological interventions, such as mowing [16], tillage, and specific cultivation practices for weed control [15], such as herbicide application or cover crop planting. Alternate mowing can be a useful practice to enhance natural antagonists against pests in vineyard inter-rows, although for optimal results mowing must be precisely timed to accord with the life cycles of the antagonists [16]. The soil of vineyards can be permanently grassed or sown with temporary cover crops [8]. Nevertheless, many farmers still opt for spontaneous vegetation from the soil seed bank instead of seeding cover crop mixtures [17,18]. In the temperate Mediterranean environment, vineyard rows are often sown with a mixture of grasses and legume species. For permanent grassing, a mixture is preferred to a monostand as it has better persistence, and provides greater genetic and functional diversity, hence greater resilience against biotic and abiotic stresses [19,20]. However, inter-row vegetation is not always effective, as the success of permanent vegetation cover is contingent on local environmental conditions. To ensure plant persistence and stability, species should be chosen according to their adaptability to local environmental conditions and specific management practices [3]. The persistence of inter-row vegetation is mainly related to its ability to compete with weeds under low-input management conditions [3].

The vineyard management makes this environment different from shrubland or forest. The variations in temperature due to the presence of shaded areas, in soil nutrient status, and in root competition typical of shrublands add to soil physical characteristics as a result of tractor traffic, which compacts the soil, vineyard inter-row mowing, and grapevine pruning management. To understand the adaptability of the herbaceous species used in vineyard inter-rows to microenvironmental conditions, a two-year study was carried out to assess their persistence in a hillside vineyard in northern Italy. It has been demonstrated that plants selected for vineyard inter-row planting, as well as spontaneous species, have different persistence according to vineyard-scale environmental conditions, including management practices [3,21,22]. Our hypothesis was that inter-row vegetation composition changes at an inter-row level driven by microenvironmental conditions that can be assessed through soil compaction, soil moisture, and soil temperature. However, the selection of shade or soil-compaction tolerant species could not be enough to ensure persistence in inter-row vegetation composition.

## Materials and methods

### Experimental site and design

The study was conducted at the Il Mottolo farm in Arquà Petrarca (N 45°15’09, E 11°42’09), northeastern Italy, from October 2018 to October 2020. The farm is an organic, non-irrigated commercial vineyard at 35 meters above sea level on a 15% slope that was planted with *Vitis vinifera* L. cv. Cabernet Sauvignon in 2004 (14 years old). The area is characterized by parent rock of a calcareous and marl type. The soil particle composition is 39.6% sand, 41.7% silt, and 18.7% clay with 13% organic matter, a total N content of 1.0 mg g^–1^ (combustion method), an Olsen P content of 4.5 mg kg^–1^, and an exchangeable K content of 132.6 mg kg^–1^ (buffered BaCl_2_ method). The area has a humid subtropical climate with an annual precipitation of 963 mm and an average air temperature of 13.5 °C (Table 1).

**Table 1:** Monthly mean air temperatures and precipitation during the study period, and long-term averages (1994-2020) at Arquà Petrarca, northeastern Italy.

	Air temperature (°C)				Precipitation (mm)			
Month	2018	2019	2020	1994-2020	2018	2019	2020	1994-2020
January	5.8^†^	2.8	4.7	3.3	28	17	18	49
February	3.2	7.5	7.9	4.9	70	56	10	64
March	6.6	10.6	9.2	8.9	163	6	69	68
April	15.9	12.8	14.6	12.8	43	206	12	95
May	19.0	13.9	17.9	17.4	84	224	41	96
June	22.4	24.8	21.0	21.7	91	33	102	80
July	24.7	24.8	24.2	23.9	90	106	76	70
August	24.9	24.7	24.4	23.6	142	38	192	71
September	20.6	19.5	20.2	18.8	114	94	38	88
October	15.6	15.3	13.2	13.8	121	109	133	98
November	9.9	9.7	9.2	8.5	96	273	15	111
December	3.9	6.2	5.2	4.0	17	101	193	74
Annual	14.4	14.4	14.3	13.5	1058	1263	899	963

† Data from Regional Agency weather station [3,23].

The present study reports a specific analysis of vegetation cover behavior within the inter-row. It is part of a larger experiment, which includes the influence of vegetation types on vine performance, grape composition, soil compaction, and erosion, as previously published [3]. The field trial is described in Pornaro et al. [3] and consisted in comparing seven types of inter-row cover: tilled soil (BS), two mixtures of cool-season grasses and a legume (M1 and M2), three blends of cool-season grasses (S1, S2, and S3), and soil covered by wild species (spontaneous vegetation, SV). The two grass-legume mixtures were composed of four or five species of selected low-growing grasses and *Trifolium repens* (M1 and M2). The grass blends were composed of three turf-type varieties of *Shedonorus arundinaceus* (S1), *Lolium perenne* (S2), and *Festuca rubra* (S3). The two selected mixtures were commonly available in the Italian market, while the species in the blends were chosen because they included in the mixtures and/or wild species present in the area. The compositions and sowing rates of each blend and mixture are reported in Table 2. All the blends and mixtures were sown in September 2018 after the grape harvest and following soil preparation and fertilization. The botanical composition of spontaneous vegetation plots was reported in Table S1. Each type of inter-row cover was cut three times per growing season (May, June, and September) using a rotary motor machine set at a height of 6 cm, either with or without mulching (M or NM). After the various types of inter-row cover had been cut, the exposed soil in the BS was plowed.

**Table 2:** Species and cultivars of the three grass blends and the two grass-legume mixtures sown in the vineyard inter-rows at Arquà Petrarca, northeastern Italy. The sowing amount is reported as weight per unit surface area, followed by the percentage on seed weight in brackets.

Blend (S)/Mixture (M)	Species	Cultivar	Sowing amount (kg ha^-1^)
S1	*Schedonorus arundinaceus*	Rhambler SRP Olympic Gold Lexington	5 (33%) 5 (33%) 5 (33%)
S2	*Lolium perenne*	Ecologic Presidian New Orleans	5 (33%) 5 (33%) 5 (33%)
S3	*Festuca rubra*	Maxima Reverent Kent	5 (33%) 5 (33%) 5 (33%)
M1	*Lolium perenne* *Festuca rubra* *Poa pratensis* *Trifolium repens*	Stefani Reverent Balin G.Huia	4.5 (45%) 4.0 (40%) 0.8 (8%) 0.7 (7%)
M2	*Festuca rubra* *Poa pratensis* *Lolium perenne* *Festuca ovina var. duriuscula* *Trifolium repens*	Gondolin Balin Option Ridu Winterwhite	5.0 (50%) 3.0 (30%) 1.0 (10%) 0.5 (5%) 0.5 (5%)

**Fig 1:**
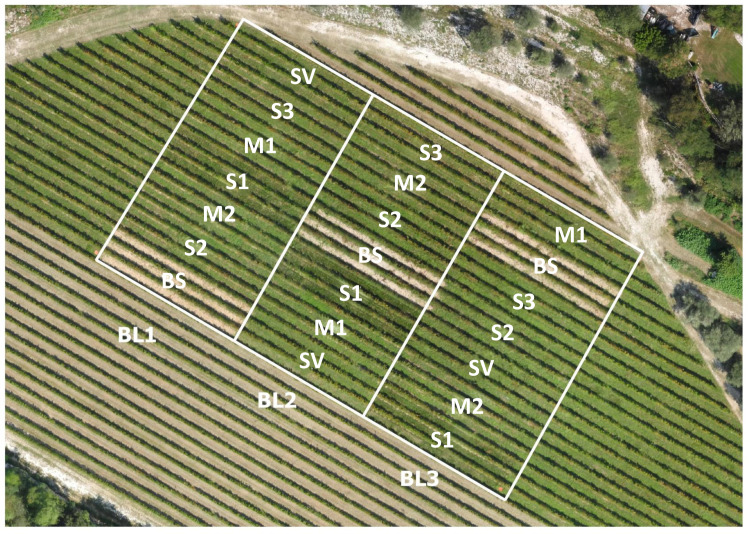
Plot arrangement for the field trial in a vineyard in Arquà Petrarca (Padua, northeastern Italy, N 45°15’09, E 11°42’09, 35 m a.s.l.) (image acquired by F. Tonon in September 2020). S1, S2, and S3 =  grass blends, M1 and M2 =  grass-legume mixtures, BS =  tilled soil, SV =  spontaneous vegetation, BL1-BL3 =  replicates [3].

The trial had a strip-plot design with three replicates. Each whole plot contained a type of inter-row cover and was split in sub-plots by mulching or no mulching (Fig 1). A vegetation plot consisted of three adjacent inter-rows (each 1.30 m x 24 m), and mulch was laid in a 12-meter-wide strip across all the plant species of each replicate. The central inter-row was the test area.

No weed control was carried out before or during the trial. At sowing and in March 2019 and 2020, fertilizer manure pellets (4 N - 4 P_2_O_5_ - 4 K_2_O) were distributed at a rate of 70 kg ha^-1^ of N. Vine management, including pesticide treatment, disease control, and other cultivation practices, such as vine canopy management, was carried out in accordance with industry standards. Most of the farming operations in the vineyard were carried out using tracked or tired tractors carrying or towing agricultural implements; these passed between the vine rows three times during the inter-row growing season in 2019 (May, June, and September) and twice in 2020 (May and September) to cut the inter-row vegetation, nine times from March to September for vine pest and disease control, and once at the beginning of October for the grape harvest. The tractor was a New Holland TD4030F with a 57kw engine, Goodyear front (280/70 R16) and rear tires (360/70 R24), and weighing 2.67 tons.

### Botanical surveys and soil measurements

Botanical surveys of each sub-plot were conducted in August of both experimental years (2019 and 2020). Vertical steel needles were placed at 50-centimeter intervals along 10-meter linear transects to identify and record plant species named based on Pignatti [24]. If the steel needle was not touched by any plant species, it was recorded as bare soil. Daget and Poissonet’s equation [25] was used in each survey to compute species relative abundances or bare soil abundance. Due to the sub-plot vegetation homogeneity, in 2019 two linear transects were placed on the two diagonals of the sub-plot, while in 2020 three linear transects per sub-plot were placed parallel to the rows: one along each tire track, and one in a line parallel to the tire track, but in the middle of the sub-plot. The data obtained were used to create species matrices for analysis. Species not present in the trial mixtures or blends (recorded in 80% of the surveys) and having mean relative abundances (average value over all surveys) greater than 10% were considered weed species. Soil compaction was assessed in October 2019 and 2020 by measuring the maximum soil penetration resistance (kPa cm^-2^) at a depth of 0-10 cm five times along each linear transect using a hand-pushed soil cone penetrometer with a cone size of 19 mm (Eijkelkamp Agrisearch Equipment, Giesbeek, The Netherlands). Soil moisture (volumetric water content) and soil temperature were measured five times in August 2020 along each linear transect using the POGO TurfPro (Stevens Water Monitoring Systems, Inc.; Portland, OR, USA) equipped with 5.6 cm metal rods.

### Data analysis

Canonical correspondence analyses (CCAs) were used to investigate the effects of inter-row cover types, mulching (mowing with or without mulching), and transect position (center - C, southwest - SW, northeast - NE) on the plant community composition. A linear mixed model was built to test the effect of type of inter-row cover (M1, M2, S1, S2, S3, SV, BS), mulching, transect position, and their interactions on parameters measured in 2020 (relative abundance of seeded species, relative abundance of weed species, soil moisture, soil temperature, and soil compaction). Main plots within blocks were included as random effects to account for the clustering of observations and ensure the independence of the model residuals. The normality and homoscedasticity of the residuals were determined by graphical analysis. Fisher’s protected LSD test at a 0.05 level of probability was used to identify significant differences between means for significant variables. The data were analyzed in R version 4.3.2 [26] with the following packages: vegan for multifactorial analysis, nlme for fitting mixed models with repeated measurements, and multcomp for post-hoc comparisons.

## Results

### Vegetation characteristics

**Fig 2:**
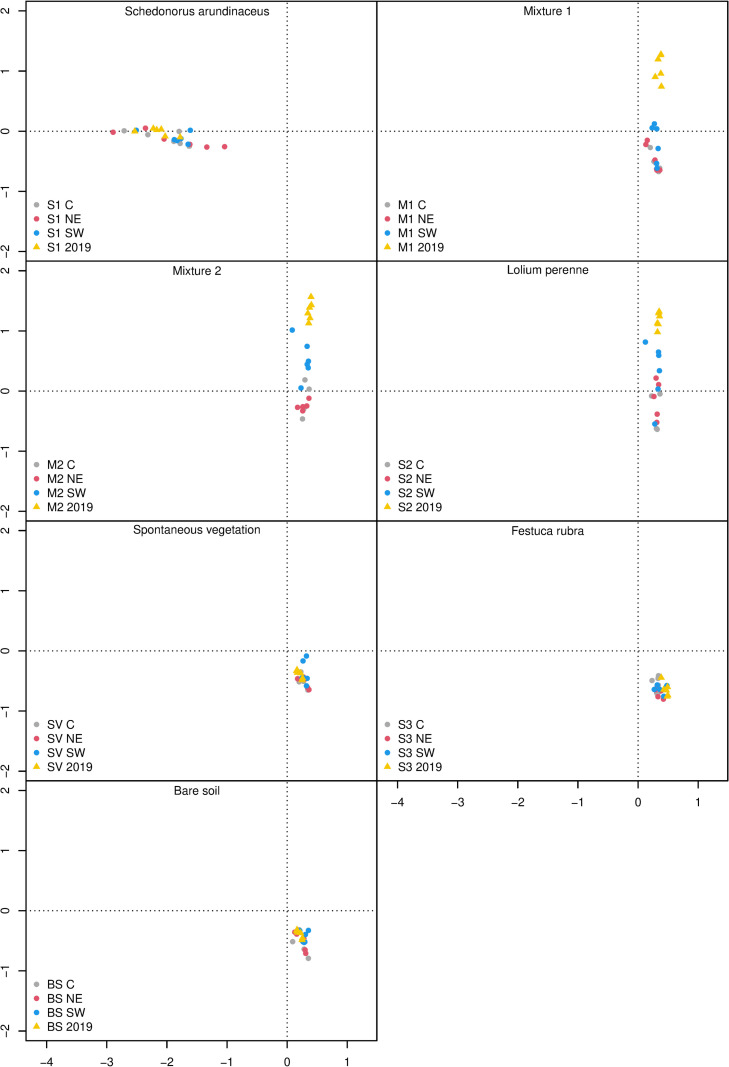
Results of the canonical correspondence analysis of vascular plant species. Triangles indicate surveys conducted in 2019, circles surveys in 2020. M1 =  grass-legume mixture 1, M2 =  grass-legume mixture 2, S1 =  *Schedonorus arundinaceus* blend, S2 =  *Lolium perenne* blend, S3 =  *Festuca rubra* blend, SV =  spontaneous vegetation, BS =  tilled soil; C =  center, NE =  northeast, SW =  southwest.

**Fig 3:**
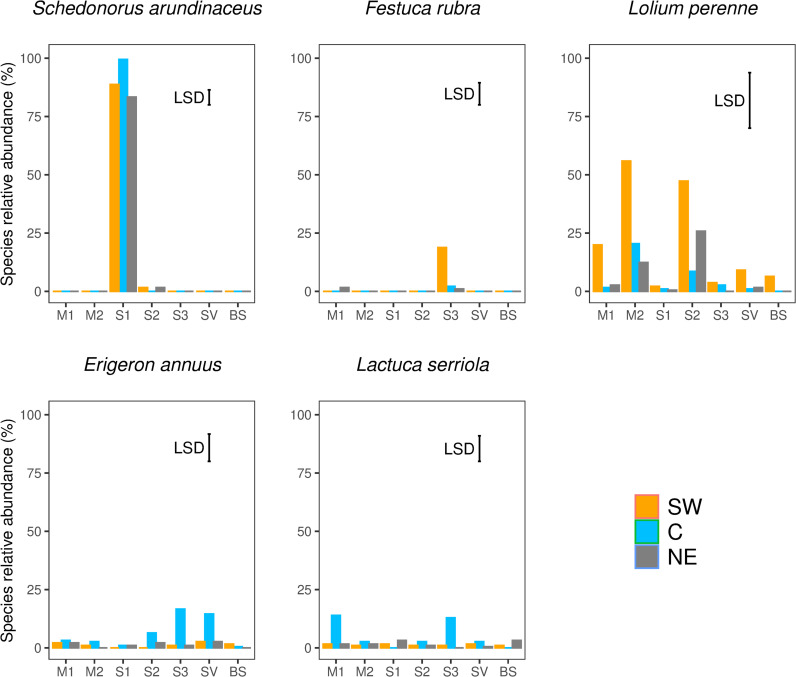
Relative abundances of the seeded species (*Schedonorus arundinaceus*, *Festuca rubra*, and *Lolium perenne*) and the weed species (*Erigeron annuus* and *Lactuca serriola*) recorded in 2020, as affected by the type of inter-row cover and transect position within the inter-row (C =  center; NE =  northeast; SW =  southwest). M1 =  grass-legume mixture 1, M2 =  grass-legume mixture 2, S1 =  *Schedonorus arundinaceus* blend, S2 =  *Lolium perenne* blend, S3 =  *Festuca rubra* blend, SV =  spontaneous vegetation, BS =  tilled soil. Error bars represent the results of the LSD test for comparing means at a 0.05 probability level.

The CCA showed that the vegetation in the *S. arundinaceus* plots (S1) differed from all the other plots in both 2019 and 2020 (Fig 2). Within the S1 plots, only the NE transect position exhibited a slight variation in botanical composition. The M1, M2, and S2 plots had similar compositions in 2019, but in 2020 they underwent a shift to a botanical composition that was similar to the SV plots, with the exception of the SW transects of M2 and S2 (Fig 3). The botanical composition of S3, BS, and SV was stable throughout the study period, with slight differences between the transect positions. Permutation test on CCA displayed a significant effect of types of inter-row cover and transect position (data not shown).

A significant interaction between type of inter-row cover and transect position was found for *S. arundinaceus*, *F. rubra*, and *L. perenne* within the seeded species, and for *Erigeron annuus* and *Lactuca serriola* within the weed species (Table 3). The effect of type of inter-row cover was significant for all species except *Festuca ovina*, while the effect of transect position was significant for *S. arundinaceus*, *L. perenne*, and *T. repens* within the seeded species, and for *C. dactylon*, *E. annuus*, *P. lanceolata*, and *S. italica* within the weed species (Table 3).

**Table 3:** Results of the analysis of variance of the effects of type of inter-row vegetation (Ty; M1 =  grass-legume mixture 1, M2 =  grass-legume mixture 2, S1 =  *Schedonorus arundinaceus* blend, S2 =  *Lolium perenne* blend, S3 =  *Festuca rubra* blend, SV =  spontaneous vegetation, BS =  tilled soil), mulching (Mu; mowed aboveground biomass released and not released), transect position within the inter-row (Tp; C =  center, NE =  northeast, SW =  southwest), and their interactions on the relative abundances of seeded and weed species and on soil characteristics (compaction, moisture, temperature) recorded in 2020.

	Parameter	Ty	Mu	Tp	Ty x Mu	Ty x Tp	Mu x Tp	Ty x Mu x Tp
Seeded species	*Schedonorus arundinaceus*	**<0.001**	0.79	**0.03**	0.99	**<0.001**	0.10	0.11
	*Festuca ovina*	0.43	0.42	0.41	0.43	0.46	0.41	0.46
	*Festuca rubra*	**<0.001**	0.72	0.06	0.99	**<0.001**	0.84	0.99
	*Lolium perenne*	**<0.001**	0.76	**<0.001**	0.99	**<0.001**	0.13	0.62
	*Poa pratensis*	**0.04**	0.27	0.64	0.11	0.89	0.74	0.95
	*Trifolium repens*	**<0.001**	0.88	**0.01**	0.48	0.06	0.89	0.94
Weed species	*Convolvulus arvensis*	**<0.001**	0.26	0.11	0.94	0.70	0.77	0.53
	*Cynodon dactylon*	**<0.001**	0.10	**<0.001**	0.95	0.57	0.46	0.08
	*Erigeron annuus*	**0.001**	0.64	**0.006**	0.91	0.002	0.98	0.99
	*Lactuca serriola*	**0.03**	0.52	0.06	0.94	0.003	0.99	0.98
	*Plantago lanceolata*	**0.03**	0.96	**0.007**	0.98	0.63	0.41	0.79
	*Setaria italica*	**<0.001**	0.73	**0.001**	0.87	0.72	0.40	0.94
	*Setaria viridis*	**0.008**	0.48	0.06	0.75	0.79	0.91	0.95
Soil	Soil compaction	**<0.001**	0.91	**0.02**	0.12	0.98	0.73	0.91
	Soil moisture	**<0.001**	0.91	**0.02**	0.24	**<0.001**	0.39	0.62
	Soil temperature	**<0.001**	0.88	**0.01**	0.99	**0.008**	0.84	0.99

*Schedonorus arundinaceus* was recorded only in the plots where it was seeded (S1), and had a higher relative abundance in the C transect position than in the SW and NE (Fig 3). Similarly, *F. rubra* was recorded only in the S3 plots, but its relative abundance was higher in the SW than in the other transect positions, and not significantly different from the other types of inter-row cover in the C and NE transect positions (Fig 3). *L. perenne* was recorded in all types of inter-row cover, with higher relative abundances in M2 and S2. Within the M2 plots, its relative abundance was greater in the SW than in the other transect positions, while within S2, it was greater in the SW than in the C transect position (Fig 3). Regarding weed species, *E. annuus* and *L. serriola* had greater abundances in the S3 C transect than in the other type of inter-row cover and the other transect positions, while *L. serriola* had greater abundance in the C transect position in both the S3 and M1 type of inter-row cover (Fig 3).

Regarding the individual effects of type of inter-row cover on the seeded species, *P. pratensis* had the highest abundance in S2, while *T. repens* had higher abundances in S3 than in S1 and BS (Table 4). The weed species had lower relative abundances in S3 than in the other type of inter-row cover, especially *Convolvolus arvensis* and *C. dactylon* (Table 4). In general, higher relative abundances were recorded in BS, especially for *C. arvensis*, *S. viridis*, and *Sorghum halepense* (Table 4). Regarding the individual effects of transect position, the highest abundances of *L. perenne* and *T. repens* were in SW, while the relative abundance of *S. arundinaceous* was higher in C than in NE (Table 5).The highest abundances of *C. dactylon* were in NE and the lowest in C, while *P. lanceolata* and *S. italica* had the highest abundances in C (Table 5).

**Table 4:** Effects of type of inter-row cover (M1 =  grass-legume mixture 1, M2 =  grass-legume mixture 2, S1 =  *Schedonorus arundinaceus* blend, S2 =  *Lolium perenne* blend, S3 =  *Festuca rubra* blend, SV =  spontaneous vegetation) and tilled soil (BS) on seeded species relative abundance (*Poa pratensis* and *Trifolium repens*), weed species relative abundance (*Convolvulus arvensis*, *Cynodon dactylon*, *Plantago lanceolata*, *Setaria italica*, *Setaria viridis,* and *Sorghum halepense*), bare soil abundance, and soil compaction (kPa cm^-2^) in 2020.

Species	M1	M2	S1	S2	S3	SV	BS
*Poa pratensis*	0.18 b^1^	0.36 b	0 b	4.12 a	0.36 b	0.36 b	0 b
*Trifolium repens*	6.81 abc	6.45 abc	4.30 bc	9.86 ab	12.76 a	7.53 abc	0.54 c
*Convolvulus arvensis*	15.59 ab	12.19 ab	10.22 b	15.23 ab	6.27 b	10.93 b	21.86 a
*Cynodon dactylon*	50.90 b	34.23 b	14.87 c	33.87 b	40.68 b	72.58 a	44.44 b
*Plantago lanceolata*	6.99 ab	4.66 ab	3.41 ab	11.47 a	5.56 ab	9.14 ab	1.08 b
*Setaria italica*	50.36 a	36.38 ab	17.74 b	32.08 ab	44.27 a	39.25 a	33.51 ab
*Setaria viridis*	9.68 ab	6.45 abc	0 c	10.40 ab	15.78 a	1.08 bc	13.26 a
*Sorgum halepense*	13.98 a	7.35 ab	4.12 b	3.94 b	10.04 ab	10.57 ab	11.65 a
Bare soil	2.69 b	5.56 ab	4.12 b	3.94 b	2.51 b	2.33 b	13.80 a
Soil compaction	54.67 bc	66.06 bc	65.22 b	59.94 bc	58.00 bc	89.11 a	44.11 c

^1^Mean values with the same letters in the same row indicate statistically non-significant differences according to an LSD test at a 0.05 probability level.

**Table 5:** Effects of transect position in the inter-row (SW =  southwest, C =  center, NE = northeast) on seeded species (*Schedonorus arundinaceus*, *Lolium perenne*, and *Trifolium repens*) relative abundance, weed species (*Cynodon dactylon*, *Plantago lanceolata*, *Setaria italica*) relative abundance, and soil compaction (kPa cm^-2^).

Specie	SW	C	NE
*Schedonorus arundinaceus*	12.90 ab	14.21 a^1^	12.14 b
*Lolium perenne*	20.66 a	5.07 b	6.15 b
*Trifolium repens*	11.06 a	4.23 b^1^	5.38 b
*Cynodon dactylon*	43.32 b	25.73 c	55.91 a
*Plantago lanceolata*	2.84 b	11.21 a	4.07 b
*Setaria italica*	28.19 b	50.15 a	30.34 b
Soil compaction	63.34 a	51.73 b	60.23 a

^1^Mean values with the same letters in the same row indicate statistically non-significant differences according to an LSD test at a 0.05 probability level.

### Soil characteristics

**Fig 4:**
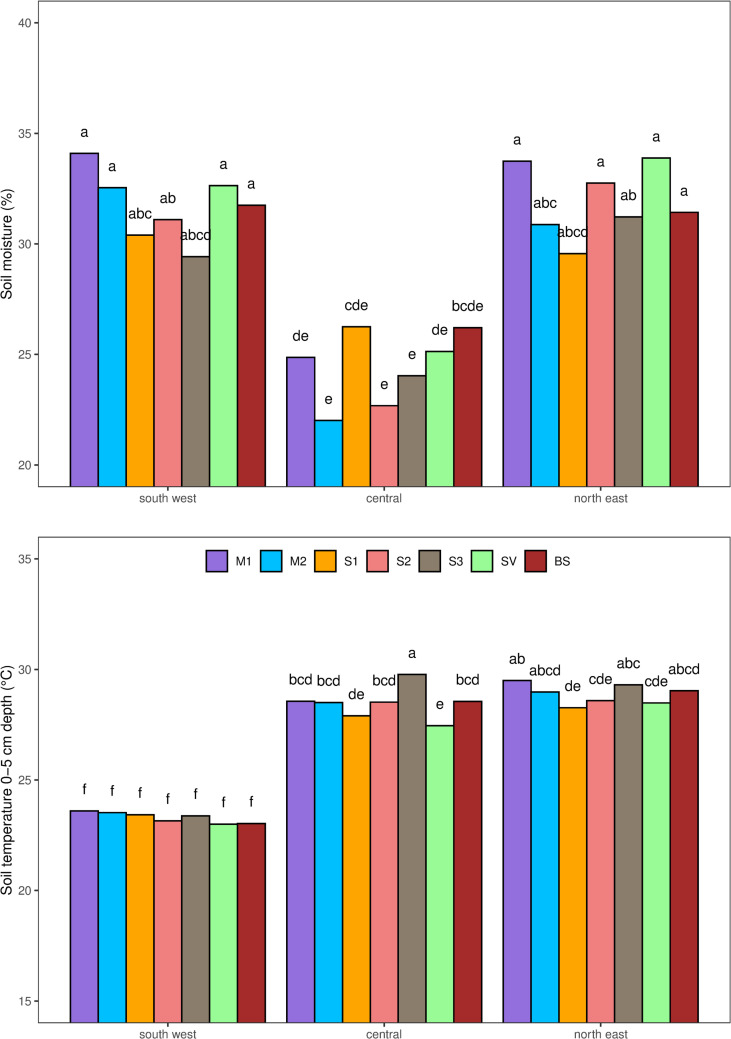
Soil moisture (upper chart) and soil temperature (lower chart) as affected by type of inter-row cover (M1 =  grass-legume mixture 1, M2 =  grass-legume mixture 2, S1 =  *Schedonorus arundinaceus* **blend, S2** **=** ***Lolium perenne*** **blend, S3** =  ***Festuca rubra*** **blend, SV** **= ** spontaneous vegetation), tilled soil (BS), and transect position within the inter-row (central, north east, south west). Bars with the same letters indicate statistically non-significant differences according to an LSD test at a 0.05 probability level.

Soil compaction was affected by the individual effect of type of inter-row cover and by the effect of transect position (Table 3). It was lower in BS (44.11 kPa cm^-2^) than in SV (89.11 kPa cm^-2^) and S1 (65.22 kPa cm^-2^) (Table 4), while the central position displayed lower value (51.73 kPa cm^-2^ in C, against 63.34 in SW and 60.23 in NE) (Table 5). Soil moisture was generally lower in the C than the other transect positions, except in S1 where no differences were found (Fig 4). Soil temperature was the same in the SW transect position of all types of inter-row cover, and it was lower than the temperature in the C and NE transect positions (Fig 4). In the C transect position, soil temperatures were higher in S3 than in the other types of inter-row cover, and lower in SV than in the other type of inter-row cover except for S1. In the NE transect position, soil temperatures were lower in S1 than in M1 and S3.

## Discussion

The results suggested differences in botanical composition at inter-row level. After two years, the botanical composition of the inter-row vegetation in the plot seeded with *F. rubra* was similar to the spontaneous vegetation (Fig 2). However, in the southwest inter-row section, where the soil temperature was lower (Fig 4), *F. rubra* comprised 22% of the botanical composition compared with 4% in the central and northeast sections (Fig 3), confirmation of the low potential adaptability of this species to the high summer temperatures in this environment [27–29]. In the plots seeded with mixtures 1 and 2, and with *L. perenne* the composition of the vegetation tended slightly toward the spontaneous, but the shift was less pronounced in the southwest section of the inter-row, especially in the plots seeded with mixture 2 and with *L. perenne* (Fig 2). These similarities suggest that it was *L. perenne* that had driven the shift in the botanical composition of the plots seeded with the two mixtures. Although the seeding rate of *L. perenne* was higher in M1 than in M2 (Table 2), after two years of study its relative abundance was higher in the southwest section of the inter-row of the M2 plots (Fig 3), which could be attributed to the different *L. perenne* cultivars in the two mixtures having different levels of environmental adaptability. Indeed, *L. perenne* has often been held to have inadequate drought tolerance mechanisms [30]. Drought resistance mechanisms of grass species fall into the categories of drought avoidance (or desiccation avoidance), drought tolerance, and drought escape [31–33]. *Loilium perenne* is generally classified as having null drought avoidance or escape capacity, poor drought tolerance mechanisms [34], and poor capacity to maintain metabolic functions at reduced internal water potential [31,32,35,36]. There may be relevant differences among cultivars, especially among turf-type genotypes [37], although no specific information is available concerning the drought stress tolerance of the cultivars used in our mixtures. However, the greater presence of *L. perenne* in the southwest position of the M2 inter-rows may also suggest its better competition capacity compared to fine fescues (*F. rubra* and *F. ovina*). In fact, seed composition of M2 was dominated by fine fescues, which have poor tolerance to high temperatures and may be replaced by a more competitive species as *L. perenne* [38]. In general, the poor performance of M2 and the S3 blend could be related to the relatively poor adaptability of *F. rubra* to the local environment (Fig 3 and Pornaro et al. [3]). The composition of the vegetation in the plots seeded with *S. arundinaceous* was more stable than the other type of inter-row cover (Fig 2), and contained a generally lower percentage of weed species (Table 4). However, the percentage of *S. arundinaceus* was lower in the southwest and northeast sections of the inter-row (Fig 3) where the canopy was damaged by the tractor passing. This species is known to be robust and to have high tolerance to traffic [39] ad for this reason its relative abundance was great also on the tire tracks, but it is characterized by a low recovery capacity due to its bunch-type growth habit [40,41], that probably affected its abundance on the tire tracks.

The weeds recorded in this study were warm-season species, which benefit from high summer temperatures [42–44]. Some species such as *E. annuus*, *L. serriola*, and *Setaria* spp., are annual or biannual species, while others, such as *C. dactylon*, are rhizomatous species. Although they will grow anywhere, these species seem to have a preference for certain type of inter-row cover (Table 4). As expected, in the inter-rows with tilled soil, we found a high percentage of weed species. This confirms that continuous soil tillering favors annual species and species propagating by rhizomes [45]. Furthermore, since the botanical composition of the tilled soil (Fig 2 and Table 4) can overlap with that of the other type of inter-row cover, according to other studies [43,44], we can assume that seeded vegetation that provides full cover is effective against weed species. We generally found high abundances of weed species, especially *E. annuus*, *L. serriola*, *C. arvensis*, and *Setaria* spp., in the inter-rows where *F. rubra* was seeded (Fig 3, Table 4). In contrast, the plots with fewer weed species were those seeded with *S. arundinaceous*. As observed by Pornaro et al. [3], in the same trial, in spring 2019, the green cover percentage of plots seeded with *F. rubra* was lower than other inter-row vegetation covers, while in summer 2019 it was the highest in plots seeded with *S. arundinaceous*. The green cover also remained lower in plots seeded with *F. rubra* than with *S. arundinaceous* during summer 2020 [3]. Considering that the botanical composition was stable throughout the study period for plots seeded with *S. arundinaceous* but less stable for plots seeded with *F. rubra* [3], it follows that the lack of canopy found in spring 2019 was filled by weed species in summer 2019 and 2020.

As also described by [3], we found that the mulching practice did not affect the botanical composition and weed species abundances (Table 3), and that the botanical composition varied two years after seeding according to the type of vegetation. However, we also found that some species remained in or spread to particular sections of the inter-row. *Lolium perenne* was more abundant in the southwest and northeast sections of the inter-row, where there was greater soil compaction (Table 5), probably because its high tolerance to traffic and ability to survive in compacted soils gives it a competitive edge over other species in this condition [46,47]. Similarly, *C. dactylon* was more abundant in the shadier southwest and northeast sections of the inter-row, even though it does not generally tolerate shade [48,49]. In contrast, *S. italica* and *P. lanceolata* were less abundant in the southwest and northeast sections, probably due to high potential competition from *C. dactylon* [48,50,51].

## Conclusion

The study revealed changes in botanical composition in the inter-row after two years of study. These changes reflect species behavior and adaptability to microenvironmental conditions. Plots seeded with *S. arundinaceus* were able to maintain a stable composition owing to the ability of this species to successfully compete with weeds. Other species, such as fine fescues, displayed less competition capacity with a consequent shift of the botanical composition which not only tends towards spontaneous vegetation but also results in a sparse canopy easily invaded by weed species. Tractor use and grapevine shade modify the microclimate in the inter-row and species, both sown and weed, find more suitable microhabitats where they are competitive against other species. These findings highlight that species selection plays a crucial role in inter-row vegetation persistence, and the choice should also take into account microclimate variability at the inter-row level.

## Supporting information

Table S1 Relative abundances of plant species (averaged over 3 replicates) recorded in the botanical surveys conducted in August 2019 and August 2020 in plots with spontaneous vegetation. (XLSX)
